# Pennsylvania State Veterinary Medical Association

**Published:** 1892-04

**Authors:** Louis Olry Lusson

**Affiliations:** Secretary, Per Revis. of W. Horace Hoskins


*Recording Secretary.*

---

*Pennsylvania State Veterinary Medical Association.*—The annual meeting of the Pennsylvania State Veterinary Medical Association was held in the Hall of the College of Physicians, Philadelphia, on Tuesday, March 8, 1892. The meeting was called promptly to order at 10 A. M. by President Kooker.

On roll call the following members responded to their names: Drs. Kooker, Thomas B. Rayner, Gladfelter, Lusson, Ridge, James B. Rayner, Glass, Hoskins, Zuill, George B. Rayner, Harger, Webster, Hart, Keil, John B. Raynor, Welker, George, Good, Kller, Knight, Custer, J. C. Michener, Collins, Robert Tonuad, Rectenwald, Meyer, Keelor, Diemer, Kuhn, Smith, Williams, Schrieber, Ross, Wemtz, Bridge, Megill, Reinhart and Weber.

As delegates, Dr. W. B E. Miller, from the Veterinary Medical Association of New Jersey; Dr. A. T. Sellers, from the Keystone Veterinary Medical Association; Dr. W. W. Ridge, representing the Philadelphia Veterinary Society.

As visitors, Dr. John Marshall, Dean of Department Veterinary Medicine, University of Pennsylvania; Dr. William Dougherty, of the Maryland Veterinary Association; Dr. E. O. Shakespeare, of the Medical Department of the University of Pennsylvania; Drs. Seuseman, Oyler, Edwards, Bloom, May, Bartholomew, Tagg and Pearson; also, Messrs. Walters and Teufel.

The election of officers resulted in the following choice: President, W. Horace Hoskins, Philadelphia; First Vice-President, Thomas B. Rayner, Chestnut Hill; Second Vice-President, R. G. Webster, Media; Third Vice-President, Z. S. Keil,

Penlasia; Recording Secretary, Robert Gladfelter, Philadelphia; Corresponding Secretary, W. H. Ridge, Trevose; Treasurer, John R. Hart, Philadelphia; Board of Trustees, W. S. Kooker, W. L. Zuill, James B. Rayne., J. Curtis Michener, S. J. J. Harger.

The Board of Trustees made the following recommendations, which were unanimously adopted: For Honorary Membership, Prof. R. S. Huidekoper; for Regular Membership, Dr. Leonard Pearson, Philadelphia, Graduate Veterinary Department, University of Pennsylvania; Dr. B. F. Seuseman, Philadelphia, Graduate Veterinary Department, University of Pensylvania; Dr. Wm. Tagg, Philadelphia, Graduate Veterinary Department, University of Pennsylvania; Dr. J. C. Bartholomew, Philadelphia, Graduate Veterinary Department, University of Pennsylvania; Dr. W. T. Edwards, Clearfield, Graduate Veterinary Department, University of Pennsylvania; Dr. J. H. Oyler, Harrisburg, Ontario Veterinary College. Unfavorably recommended: Dr. Lewis I. Bloom, non-graduate of Curwensville, Pa.

Recommended that the officers issue application blanks at once, and that at least three be sent to each member.

Charges pending against Dr. G. Meyer, of Allegheny, Pa., were laid over until the September meeting.

Committee reports being next in order, Dr. W. Horace Hoskins, Chairman of Legislative Committee, reported that prosecutions were being conducted in Allegheny County only, and on the decisions gained there would rest the future actions of the Committee in further testing the law. He reported but two additional registrations in Philadelphia County since his report in September, and but few in other counties of the State heard from.

Dr. W. L. Zuill, Chairman of Committee on Sanitary Science and Police, made an estimable report, fully covering the grounds of their work and reporting many interesting and valuable facts and statistics.

At this hour the President extended a hearty invitation to all those present to attend the meeting of the Keystone Veterinary Medical Association in the evening, in the same room, to listen to a paper by the Hon. John S. McKinley on warranty and soundness, and the laws applicable to the sale and purchase of horses in Pennsylvania.

Dr. W. Horace Hoskins, Secretary of the United States Veterinary Medical Association, gave a general invitation to all to be present at the next meeting in Boston in September, and the international meeting in Chicago in 1893.

The meeting then adjourned until 2 P. M. to partake of a bounteous lunch tendered the Association and all present by the Department of Veterinary Medicine of the University of Pennsylvania. It was served in excellent style and unlimited quantity at Boothby's restaurant, and was most thoroughly enjoyed and appreciated by all present. A hearty and sincere vote of thanks was extended the Department by the Association for its kindness and generosity.

The following members were dropped from the rolls for non-payment of fees and dues: Drs. L. E. Wheat, A. Maurise, W. B. Montgomery, W. M. Brodhead and C. E. Bridge.

Under the head of new business, Dr. Harger proposed that our annual meetings be extended to two days, in order to more thoroughly complete our work and to enable the members to visit points of interest in the city. Dr. Hoskins seconded the motion that our next annual meeting be at least two days, which was adopted.

Under the head of communications, letters of regret were read from Prof. A. Liautard, Drs. D. E. Salmon, E. A. A. Grange, John P. Turner, B. F. King, Claude

D. Morris and Wm. H. Lowe; from members Magee, F. F. Hoffman and J. H. Timberman. An interesting communication was read from E. S. Bansticker.

A number of bills were then reported from the Secretary, Treasurer and Chairmen of Committees, all of which were ordered paid.

Papers now being in order, the President called upon Dr. S. J. J. Harger, who responded with an article upon Laryngotomy or Arytenotomy for the relief of Roarers. This paper proved to be one of the most thoroughly scientific ones ever presented to the Association. It was illustrated by an exhibition of all the instruments used in the various steps of the operation and by many fresh specimens illustrating the important points of the operation. His method of operation and treatment was compared with that of Hemings, and his success in some four cases reported, reflected great merit upon the results achieved. The paper was discussed by many of the members, and many interesting points brought forth relative to Roaring from various causes, congenital, accidental, conformation, checking, etc. It was decided to print a thousand copies of the paper and the kind offer of Mr. Tenful, instrument maker, to furnish the necessary cuts for its proper illustration was accepted with a vote of thanks.

The paper of Dr. James A. Waugh on the Veterinarian—Morally, Socially and Intellectually—was read by Dr. Alex. Glass. It was replete with good thoughts and pungent suggestions. Its caustic criticisms of the multiplication of mushroom colleges and their short courses struck a sympathetic chord in the members of the Association, and the paper was much appreciated by all present.

The report of Drs. Ridge and Formad on the so-termed Uterine Calculi, referred to at our September meeting by Dr. Keller, found in his practice, proved conclusively that they were of similar conformation to cobble stones and could not be considered in any way as having developed in the animal economy; but their presence in the three animals referred to by Dr. Keller could only be considered as a huge joke and the work of some practical joker. Similar curious bodies were reported by Drs. Miller, Rectenwald and others, placed in the uterine passages to prevent prolapsus and to deter rutting in swine.

Dr. E. O. Shakespeare, in commenting upon Tuberculosis, spoke of the uncertain value of Tuberculin in diagnosing Tuberculosis by its hypodermic injection.

Dr. W. Horace Hoskins, referring to Dr. Waugh's paper, thought the time had now arrived when the various Veterinary Associations throughout the land should take cognizance of the multiplication of imperfectly organized and equipped veterinary colleges, whose short courses and easily obtained diplomas would soon reflect great discredit upon the whole profession. He thought the mere possession of a diploma should not be a sufficient warranty of an applicant's fitness for membership in our Associations.

The new officers were then seated with appropriate remarks, and a vote of thanks extended the retiring officers for their earnest and efficient work.

Allentown was selected as the place for the semi-annual meeting in September.

An invitation by Dr. W. B. E. Miller to the meeting of the Veterinary Medical Association of New Jersey was followed by a motion for adjournment.

LOUIS OLRY LUSSON, *Secretary*,

Per Revis. of W. HORACE HOSKINS.

